# Clinical Relevance of Viable Circulating Tumor Cells in Patients with Metastatic Colorectal Cancer: The COLOSPOT Prospective Study

**DOI:** 10.3390/cancers13122966

**Published:** 2021-06-13

**Authors:** Thibault Mazard, Laure Cayrefourcq, Françoise Perriard, Hélène Senellart, Benjamin Linot, Christelle de la Fouchardière, Eric Terrebonne, Eric François, Stéphane Obled, Rosine Guimbaud, Laurent Mineur, Marianne Fonck, Jean-Pierre Daurès, Marc Ychou, Eric Assenat, Catherine Alix-Panabières

**Affiliations:** 1 IRCM, Inserm, University of Montpellier, ICM, 34000 Montpellier, France; marc.ychou@icm.unicancer.fr; 2 Department of Medical Oncology, University Medical Center of Montpellier, St. Eloi Hospital, 34295 Montpellier, France; e-assenat@chu-montpellier.fr; 3 Laboratory of Rare Human Circulating Cells, University Medical Center of Montpellier, University of Montpellier, 34093 Montpellier, France; l-cayrefourcq@chu-montpellier.fr; 4 CREEC, MIVEGEC, University of Montpellier, CNRS, IRD, 34000 Montpellier, France; 5 Biostatistiques, Nouvelles Technologies, AESIO Santé, 34394 Montpellier, France; f.perriard@languedoc-mutualite.fr (F.P.); jp.daures@languedoc-mutualite.fr (J.-P.D.); 6 Department of Medical Oncology, Institut de Cancérologie de l’Ouest, 44800 Saint Herblain, France; helene.senellart@ico.unicancer.fr; 7 Department of Oncology, Institut de Cancérologie de l’Ouest, 49100 Nantes-Angers, France; benjamin.linot@groupeconfluent.fr; 8 Department of Medical Oncology, Centre Léon Bérard, 69008 Lyon, France; christelle.delafouchardiere@lyon.unicancer.fr; 9 Department of Gastroenterology, CHU Haut-Lévêque, 33600 Pessac, France; eric.terrebonne@chu-bordeaux.fr; 10 CLCC Antoine Lacassagne, 06100 Nice, France; eric.francois@nice.unicancer.fr; 11 Department of Gastroenterology, University of Montpellier-Nîmes, Carémeau Hospital, 30900 Nîmes, France; stephane.obled@chu-nimes.fr; 12 Department of Oncology, Toulouse-Rangueil University Hospital, 31059 Toulouse, France; guimbaud.r@chu-toulouse.fr; 13 Oncology, Radiotherapy, Sainte-Catherine Institut, 84918 Avignon, France; l.mineur@isc84.org; 14 Department of Medical Oncology, Institut Bergonié, 33000 Bordeaux, France; m.fonck@bordeaux.unicancer.fr

**Keywords:** circulating tumor cells, colorectal cancer, EPISPOT assay, CellSearch^®^ system, predictive value

## Abstract

**Simple Summary:**

The analysis of circulating tumor cells (CTCs) as a “real-time liquid biopsy” in epithelial tumors for personalized medicine has received tremendous attention over the past years, with important clinical implications. In metastatic colorectal cancer (mCRC), the CellSearch^®^ system has already demonstrated its prognostic value and interest in monitoring treatment response, but the number of recovered CTCs remains low. In this article, we evaluate the early prognostic and predictive value of viable CTCs in patients with mCRC treated with FOLFIRI–bevacizumab with an alternative approach, the functional EPISPOT assay. This study shows that viable CTCs can be detected in patients with mCRC before and during FOLFIRI–bevacizumab treatment and that CTC detection at D_28_ and the D_0_–D_28_ CTC kinetics evaluated with the EPISPOT assay are associated with response to treatment.

**Abstract:**

Background: Circulating tumor cells (CTCs) allow the real-time monitoring of tumor course and treatment response. This prospective multicenter study evaluates and compares the early predictive value of CTC enumeration with EPISPOT, a functional assay that detects only viable CTCs, and with the CellSearch^®^ system in patients with metastatic colorectal cancer (mCRC). Methods: Treatment-naive patients with mCRC and measurable disease (RECIST criteria 1.1) received FOLFIRI–bevacizumab until progression or unacceptable toxicity. CTCs in peripheral blood were enumerated at D_0_, D_14_, D_28_, D_42_, and D_56_ (EPISPOT assay) and at D_0_ and D_28_ (CellSearch^®^ system). Progression-free survival (PFS) and overall survival (OS) were assessed with the Kaplan–Meier method and log-rank test. Results: With the EPISPOT assay, at least 1 viable CTC was detected in 21% (D_0_), 15% (D_14_), 12% (D_28_), 10% (D_42_), and 12% (D_56_) of 155 patients. PFS and OS were shorter in patients who remained positive, with viable CTCs between D_0_ and D_28_ compared with the other patients (PFS = 7.36 vs. 9.43 months, *p* = 0.0161 and OS = 25.99 vs. 13.83 months, *p* = 0.0178). The prognostic and predictive values of ≥3 CTCs (CellSearch^®^ system) were confirmed. Conclusions: CTC detection at D_28_ and the D_0_–D_28_ CTC dynamics evaluated with the EPISPOT assay were associated with outcomes and may predict response to treatment.

## 1. Introduction

In western countries, colorectal cancer (CRC) is one of the most frequently diagnosed cancers and a leading cause of cancer death. In Europe, an estimated 499,700 new cases occurred in 2018, and 242,500 patients died of CRC in the same year [1]. 

CRC’s high mortality rate is due to the development of distant unresectable metastases in more than 50% of patients at some point during the disease course [2]. In this setting, the current guidelines recommend the use of cytotoxic chemotherapy regimens that combine fluoropyrimidine with oxaliplatin or irinotecan and a targeted agent (bevacizumab or cetuximab/panitumumab) as first-line standard-of-care therapy, [3,4,5]. Although the RAS oncogene’s mutational status is an unquestionable marker to select patients who are unlikely to benefit from EGFR antibody therapy [6,7], robust biomarkers for predicting outcome and early treatment response are still lacking, especially for bevacizumab-based regimens [8]. 

The “liquid biopsy” has been introduced for the analysis of circulating tumor cells (CTCs) in the blood of patients with solid cancers, and many clinical trials have focused on this new approach for precision medicine over the past decade [9]. Specifically, the most aggressive tumor cells are actively released by the tumor and/or metastases in body fluids [10]. They can be isolated from peripheral blood and were the first “liquid biopsy” component investigated as a biomarker in many cancer types [9]. In metastatic CRC, the CTC prognostic value has been clinically validated using the FDA-cleared CellSearch^®^ system (www.cellsearchctc.com, accessed on 21 May 2021). Briefly, three large prospective studies demonstrated that patients with ≥3 CTCs before chemotherapy have shorter progression-free (PFS) and overall survival (OS) [10,11,12]. They also found that the CTC number remains a strong prognostic factor after a few treatment cycles and might also help monitor the treatment response. In these studies, most patients received the fluoropyrimidine–oxaliplatin combination and bevacizumab as first-line treatment. With the CellSearch^®^ system, CTC capture is based on immunoselection using antibodies against the epithelial cell surface adhesion molecule (EpCAM) [13]. However, CTCs are phenotypically heterogeneous, and some may not express epithelial markers anymore or weakly, especially if they have undergone an epithelial-to-mesenchymal transition [14,15]. Consequently, these subpopulations might not be detected by the CellSearch^®^ system, underlining the need to develop alternative approaches to improve CTC enrichment.

In this context, we developed a functional assay called the Epithelial ImmunoSPOT assay (EPISPOT) that selects viable CTCs based on the detection of specific secreted tumor-associated proteins. Therefore, EPISPOT enumerates only viable CTCs, irrespective of EpCAM expression, because this innovative technology is always combined with depletion of leukocytes [16]. Using cytokeratin-19 (CK19) as the released protein to detect CTCs in the bloodstream, we have already validated the prognostic value of functional CTCs in a prospective study with more than 250 patients with metastatic breast cancer. We found that functional CTCs are correlated with OS and could be used in combination with the CTCs detected by the CellSearch^®^ system to refine the prognostic stratification of these patients [17]. Moreover, in non-metastatic CRC, the CK19-EPISPOT assay detected more CTCs than the CellSearch^®^ system in peripheral and mesenteric blood samples from patients with treatment-naïve tumors [18].

Therefore, we carried out a prospective study, called COLOSPOT, on patients with untreated metastatic CRC, about to receive FOLFIRI (folinic acid, fluorouracil, and irinotecan) and bevacizumab as first-line therapy, to further investigate the clinical utility of viable CTCs detected with the CK19-EPISPOT assay. The objectives were to assess the prognostic and early predictive values of viable CTC enumeration and their dynamics during treatment using the CK19-EPISPOT assay and to compare the CTC detection of the CK19-EPISPOT assay and the CellSearch^®^ system (the gold standard).

## 2. Materials and Methods

### 2.1. Study Design

We carried out a multicenter prospective study named “COLOSPOT” (ClinicalTrials.gov: NCT01596790) in 11 medical centers in France. The human investigations were performed after approval by the human investigation committee Sud Méditerranée III (Ref: 2011.11.01). Patients with untreated metastatic colorectal adenocarcinoma, with measurable disease according to Response Evaluation Criteria in Solid Tumors (RECIST) 1.1, who started first-line systemic therapy with FOLFIRI–bevacizumab were eligible. Other inclusion criteria were: patients older than 18 years and an Eastern Cooperative Oncology Group (ECOG) performance status (PS) score of 0 to 2. Chemotherapy was continued until disease progression, unacceptable toxicity, or patient/investigator’s decision. Tumor response was assessed every 8 weeks during the first year of treatment and every 3 months thereafter until disease progression or for a maximum period of 2 years. Tumor response was evaluated using contrast-enhanced chest–abdomen–pelvis computed tomography images and the RECIST 1.1 criteria. All patients gave their written informed consent before inclusion.

For CTC enumeration, peripheral blood samples were drawn just before and during therapy, as follows: for the EPISPOT assay, 15 mL of blood was collected in EDTA tubes at baseline (D_0_) and at day 14 (D_14_), day 28 (D_28_), day 42 (D_42_) and day 56 (D_56_) after treatment initiation. For the CellSearch system, 10 mL of peripheral blood was collected in CellSave tubes (Silicon Biosystems-Menarini) at D_0_ and D_28_, based on the data previously reported by Cohen et al., showing that the conversion of baseline unfavorable (≥3 CTCs/7.5 mL of blood) to favorable (<3 CTCs/7.5 mL of blood) CTC profiles at 3–5 weeks is associated with significantly longer PFS and OS [10]. All blood samples were sent to LCCRH–Montpellier, where all the CTC detection experiments were processed.

### 2.2. CTC Isolation and Enumeration

All CK19-EPISPOT assays were performed at LCCRH–Montpellier. The detailed procedure of the EPISPOT assay has been previously described [16]. Briefly, within 24 h after blood collection, leukocytes were depleted with RosetteSep CTC enrichment cocktails (#15167) from Stemcell Technologies. Then, the enriched fraction was frozen in liquid nitrogen (90% fetal calf serum + 10% DMSO) and unfrozen when all samples from the same patient were obtained. The idea was to run a single CK19-EPISPOT experiment per patient, avoiding inter-assay variation during the follow-up. Enriched cells were cultured in 96-well plates (MAIPN4550, Milipore, Darmstadt, Germany), precoated with an anti-CK19 antibody (Ks19.1, Progen, Heidelberg, Germany), to capture CK19-releasing CTCs. After 48 h, wells were washed to remove cells, and CK19 molecules captured by the coating antibody were detected with a second anti-CK19 antibody (Ks19.2, Progen) conjugated to the AlexaFluor 555 fluorochrome. Single fluorescent CK19 immunospots were counted under a fluorescent microscope equipped with a camera and computer-assisted analysis (KS ELISPOT, Carl Zeiss Vision, Oberkochen, Germany). Results were expressed as the number of cells per 15 mL of blood. 

All CellSearch^®^ analyses were performed within 96 h after blood collection using the CellSearch^®^ CTC kit (7900001, Silicon Biosystem, Menarini, Bologna, Italy), according to the manufacturer’s instructions. This method enriches CTCs via positive selection with magnetic beads coated with anti-EpCAM antibodies, followed by immunofluorescence-based detection. CTCs are Pan-CK^(+)^, DAPI^(+)^, and CD45^(−)^. Results are expressed as the number of cells per 7.5 mL of blood. 

### 2.3. Statistical Analyses

Data were summarized with medians and ranges for continuous variables and frequency for categorical variables. Fischer’s exact test was used to study the correlation between CTC detection and clinical–pathological characteristics. Concordance between technologies was assessed at D_0_ and D_28_ by calculating the intraclass correlation coefficient.

PFS and OS were analyzed with the Kaplan–Meier method. Survival curves were compared with the non-parametric log-rank test (*p* ≤ 0.05 was considered significant). PFS was defined as the elapsed time from blood collection to disease progression or death from any cause. Patients who began a second-line treatment without disease progression were censored at the date of treatment switch. OS was defined as the elapsed time from blood collection to death from any cause.

Univariate and multivariate Cox proportional hazards regression models were used to obtain the unadjusted and fully adjusted hazard ratios (HRs) and 95% confidence intervals (CIs). 

Statistical analyses were performed with SAS version 9.4 (SAS Institute, Cary, NC, USA).

## 3. Results

### 3.1. Clinical and Tumor Characteristics 

**Figure 1:**
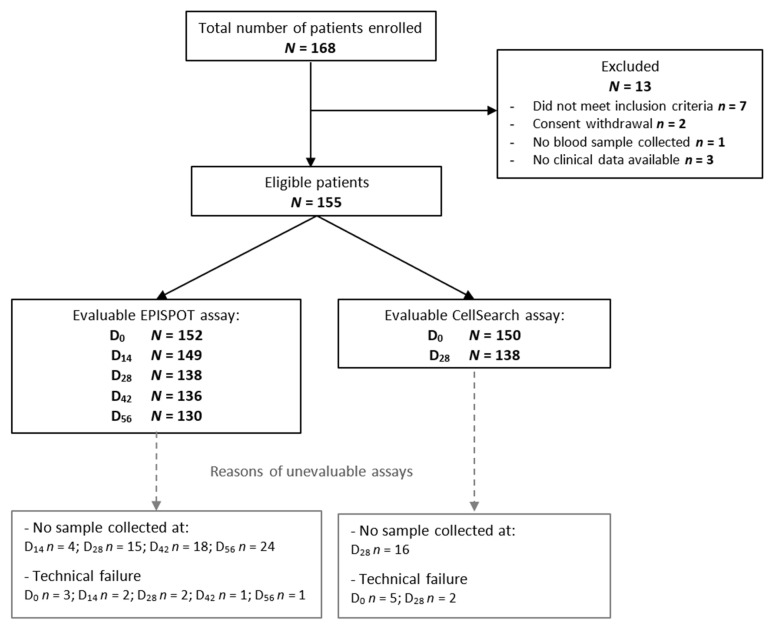
Study flowchart showing the number of included patients and the number of patients in whom CTCs could be assessed in peripheral blood samples at different time points before (D_0_) and during treatment (EPISPOT: D_14_, D_28_, D_42_, D_56_; CellSearch^®^: D_28_). Abbreviations: N, number; D, day.

Between April 2012 and September 2016, 168 patients were enrolled in the study, among whom 155 met the inclusion and exclusion criteria and were assessable. The number of patients included at each stage of the analysis and the reasons for exclusion are summarized in the study flowchart (Figure 1).

The patient and tumor characteristics are summarized in Table S1. At the time of the final analysis (July 2019), the median follow-up was 24.5 months (range, 0.99–75.04 months), and the median PFS and OS were 9.4 (95% CI, 8.1–10.2 months) and 26.2 months (95% CI, 21.3–29.8 months), respectively.

### 3.2. CTC Prevalence at Different Time Points and Correlation with Baseline Characteristics

Table S2 summarizes the results obtained with the CK19-EPISPOT and CellSearch^®^ assays at different time points. With the EPISPOT assay, 32/152 (21%) patients had ≥1 CTC/sample and 18/152 had ≥2 CTCs/sample (11.8%) at D_0_. During treatment, the number of patients with at least 1 CTC decreased to 15.4% at D_14_, 12.3% at D_28_, 9.6% at D_42_, and 11.5% at D_56_. According to the CellSearch^®^ assay, 59/150 (39.3%) and 13/138 (9.4%) patients had ≥3 CTCs/sample at D_0_ and D_28_, respectively.

The concordance between methods was low, as indicated by the Cohen Κ coefficient of 0.23 (*p* = 0.002) and 0.34 (*p* ≤ 0.0001) at D_0_ and D_28,_ respectively. 

Only CTCs detected with the CellSearch^®^ system at D_0_ (≥3) correlated significantly with some biological and clinical characteristics. Baseline performance status was worse and more patients had synchronous metastases, liver involvement, and abnormal CEA levels in the group with ≥3 CTCs/sample than in the group with <3 CTCs/sample at D_0_ (Table 1).

### 3.3. CTC Presence Correlates with PFS and OS in Patients with Metastatic CRC

**Figure 2:**
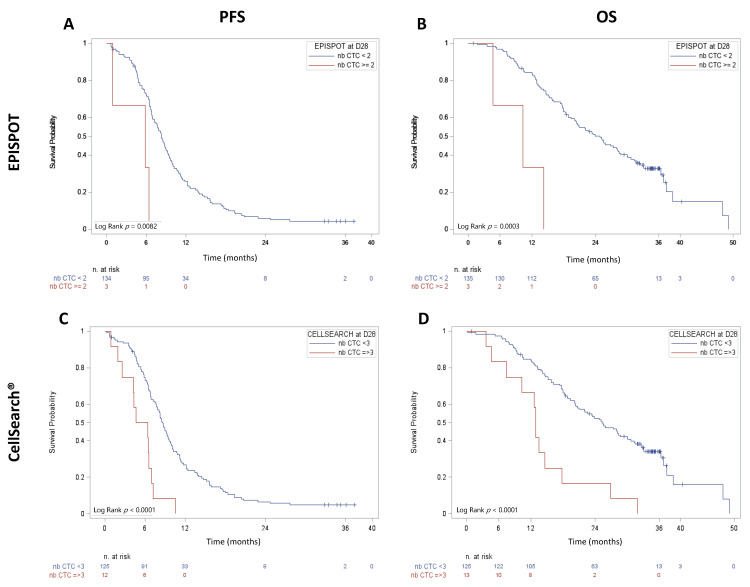
PFS and OS in patients with metastatic CRC at D_28_. CTCs were enumerated after the first two chemotherapy cycles (D_28_) with the (**A**,**B**) CK19-EPISPOT (≥2 vs. <2) and (**C**,**D**) CellSearch^®^ (≥3 vs. <3) assays.

Considering the CTC data obtained with the CK19-EPISPOT assay, the number of viable CTCs at D_28_, but not at D_0_, was significant correlated with PFS and OS (Figure 2A,B). PFS and OS were shorter in patients with ≥2 CTCs than in patients without or with only 1 CTC (median PFS = 5.82 months, 95% CI (0.92–6.37 months) vs. 8.28 months, 95% CI (7.20–9.17 months); *p* = 0.0082 and median OS = 10.28 months, 95% CI (4.63–14.26 months) vs. 24.84 months, 95% CI (20.11–28.45 months); *p* = 0.0003). Similar results were obtained for CTCs at D_42_ and OS. No prognostic correlation was observed using 1 CTC as cut-off, regardless of the sampling time (Table S3).

With the CellSearch^®^ system, at D_0_, OS was shorter in patients with ≥3 CTCs than in those with <3 CTCs (median OS = 19.1 months, 95% CI (15.57–21.59 months) vs. 37.3 months, 95% CI (26.81–44.58 months); *p* < 0.0001). Conversely, PFS was not significantly different (data not shown). At D_28_, ≥3 CTCs was associated with shorter PFS and OS compared with <3 CTCs (median PFS = 5.50 months, 95% CI (1.90–6.93 months) vs. 8.64 months, 95% CI (7.67–9.56 months); *p* < 0.0001 and median OS = 12.91 months, 95% CI (4.63–17.77 months) vs. 25.27 months, 95% CI (20.40–30.10 months); *p* < 0.0001 respectively) (Figure 2C,D).

### 3.4. CTC Kinetics between D_0_ and D_28_ Correlates with PFS and OS

**Figure 3:**
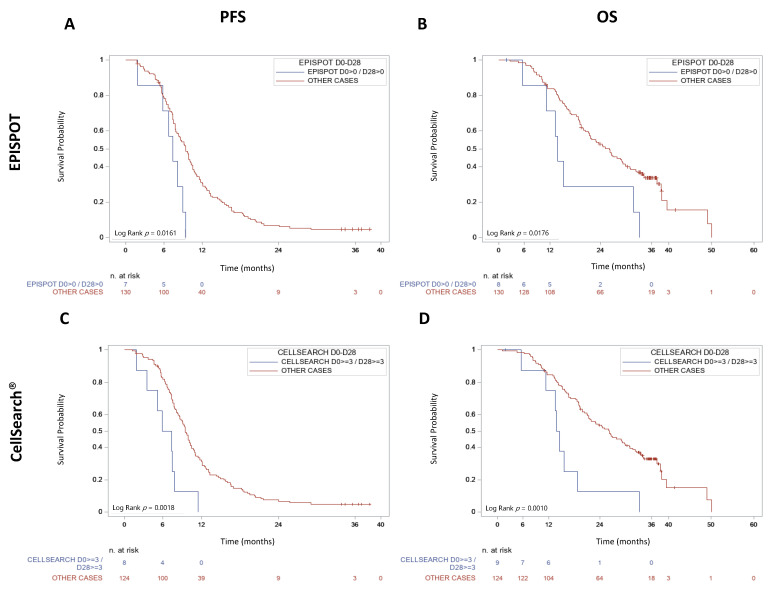
PFS and OS in metastatic CRCs according to the D_0_–D_28_ CTC kinetics. Patients were divided into two groups in the function of CTC enumeration at D_0_ and D_28_, using the (**A**,**B**) CK19-EPISPOT and (**C**,**D**) CellSearch^®^ assays.

To study the CTC kinetics between D_0_ and D_28_, patients were divided in two groups: (1) CTC-positive at D_0_ and D_28_, and (2) CTC-negative at D_0_ and D_28_ or CTC-positive only at D_0_ or D_28_. PFS and OS were significant shorter in patients in the first group, with both the CK19-EPISPOT method (median PFS = 7.36 months, 95% CI (1.84–8.97 months) vs. 9.43 months, 95% CI (8.08–10.25 months); *p* = 0.0161 and median OS = 13.83 months, 95% CI (5.55–31.63 months) vs. 25.99 months, 95% CI (20.99–29.17 months); *p* = 0.0176) and the CellSearch^®^ method (median PFS = 6.6 months, 95% CI (1.84–7.85 months) vs. 9.46 months, 95% CI (8.54–10.31 months); *p* = 0.0018 and median OS = 14.13 months, 95% CI (5.55–18.69 months) vs. 26.18 months, 95% CI (21.29–29.83 months); *p* = 0.0010) (Figure 3).

Univariate analysis confirmed that the early CTC dynamics (both assays), ECOG PS at D_0_, and BRAF mutational status were predictors of PFS and OS. A primary tumor localized to the right colon also significantly correlated with worse OS (Table 2).

In multivariate analysis, D_0_–D_28_ CTC kinetics according to the CK19-EPISPOT assay (HR 2.445, 95% CI (1.04–5.78), *p* = 0.0414) and the CellSearch^®^ system (HR 2.461, 95% CI (1.06–5.74), *p* = 0.037) remained significant predictors of PFS but not of OS (Table 3).

## 4. Discussion

More than a decade ago, it was shown that CTC enumeration is a prognostic factor in metastatic breast, prostate, and colorectal cancer [10,19,20]. In this field of expertise, it was then important to show the clinical validity of CTCs with meta-analyses of thousands of cancer patients [21] and, especially, to demonstrate their clinical utility for introducing them in daily clinical practice [22]. CTC clinical validity and utility have been reported for metastatic breast cancer; conversely, in CRCs, many key questions are still unanswered. 

To determine whether viable CTCs are clinically relevant in patients with metastatic CRC as an early criterion of response to FOLFIRI–bevacizumab treatment, we performed a prospective multicenter study in which peripheral blood samples were tested before and during treatment, with two different CTC detection technologies: (i) the EPISPOT assay to detect viable CTCs, and (ii) the FDA-cleared CellSearch^®^ system. We then determined whether the subpopulation of viable CTCs detected with the EPISPOT assay is clinically relevant for the prognosis and as an early biomarker to predict clinical outcomes after treatment initiation. We assessed the CTC count at different time points and different CTC cut-offs for the EPISPOT assay because this system is still under study. Conversely, on the basis of the work by Cohen et al., with the CellSearch^®^ system, we only tested CTCs at D_0_ and D_28_ and considered only the cut-off of ≥3 CTCs [10]. 

The studied population is representative because their OS (26.2 months) and PFS (9.4 months) are consistent with previously reported data on unselected patients with metastatic CRC treated with FOLFIRI and bevacizumab [23]. The detection of viable CTCs could be assessed in most patients during their routine follow-up at 11 centers in France, demonstrating the feasibility of this technique in clinical practice. During treatment, we found significant correlations between survival and the presence of viable CTCs (threshold: ≥2 CTCs) at D_28_ (PFS and OS) and D_42_ (only OS). Moreover, the D_0_–D_28_ CTC kinetics predicted both PFS and OS and was an independent factor of PFS by multivariate analysis. This finding confirms the clinical interest of the CTC kinetic previously assessed with ISET technology [24] or other assays [25] for early detection of poor outcomes in patients with metastatic CRC under treatment. During the last decade, the EPISPOT assay’s prognostic value has already been demonstrated in advanced breast, prostate, and head and neck cancer as well as in melanoma and non-metastatic CRC [18,26,27,28]. The prognostic value of the early kinetics of viable CTCs has already been reported in recurrent and metastatic head and neck squamous cell carcinoma [28].

According to the CellSearch^®^ system, 40% of patients had ≥3 CTCs, in line with previous studies (24–52% of untreated patients with metastatic CRC) [10,11,12,29,30]. We then confirmed that the CellSearch^®^ system, using the conventional cut-off of 3 CTCs, provides prognostic information before and early after initiation of the first line of treatment. PFS and OS were significantly shorter in patients who became or remained positive (≥3 CTCs) after 4 weeks of chemotherapy (D_28_), demonstrating that they did not benefit from therapy. 

Considering the detection of viable CTCs (EPISPOT), the number of positive patients was lower at baseline compared with the CellSearch^®^ system, and it decreased during treatment. Thus, the low number of patients with unfavorable CTC evolution according to the EPISPOT assay is a limitation of our study. As already shown in previous studies [17,26,27,28], the concordance between EPISPOT and CellSearch^®^ technologies for CTC detection was low at baseline and during treatment. This could be explained by the fact that the EPISPOT assay detects only CK19-releasing viable CTCs and not the others (e.g., apoptotic CTCs). Moreover, the enrichment and detection steps are different. The CellSearch^®^ system uses positive selection based on EpCAMs to enrich CTCs, whereas the EPISPOT assay is combined with negative selection by leukocyte depletion. In the CellSearch^®^ system, detection is based on Pan CK, DAPI, and CD45 staining of fixed CTCs. Conversely, the EPISPOT assay detects only CK19-releasing CTCs in culture. Despite this low agreement, the dynamic CTC count, which changes with both methods, remained significantly correlated with PFS in multivariate analysis, suggesting that these assays are complementary for predicting clinical outcomes during treatment. Interestingly, CTC positivity (≥3 cells) by CellSearch^®^ is correlated with surrogate markers of tumor burden ([30,31] and the present study), but not the presence of viable CTCs. This might suggest that their predictive value is not directly linked to the tumor mass changes but more to the identification of an aggressive chemotherapy-resistant subpopulation of tumor cells that are certainly at the origin of cancer progression.

Currently, we are developing a new version of the EPISPOT assay, named EPIDROP (EPIspot in a DROP), that combines EPISPOT and CellSearch^®^ strategies and might represent an ideal liquid biopsy tool. Indeed, with this new technology, we can detect the total amount of CTCs by immunostaining, as done by the CellSearch^®^ system, and also the subset of viable CTCs on the basis of their ability to secrete, shed, or release some proteins. EPIDROP might also allow the use of a larger panel of CTC biomarkers, such as VEGF monitoring during bevacizumab therapy. This innovative technology should open new avenues to detect CTCs that are relevant as prognostic and early predictive information in metastatic CRC with high specificity and sensitivity.

## 5. Conclusions

The CK19-EPISPOT assay detects viable CTCs in metastatic CRC. This prospective study shows that real-time liquid biopsy for CTC analysis could be clinically relevant in this setting, particularly to monitor the early response to FOLFIRI–bevacizumab.

## Figures and Tables

**Table 1:** Patient characteristics and correlation with CTC number. CTCs were detected with two methods: CK19-EPISPOT and CellSearch^®^.

Parameters	EPISPOT (*n* = 152)			CellSearch^®^ (*n* = 150)		
	≥1	<1	*p*-Value (Fisher)	≥3	<3	*p*-Value (Fisher)
Age						
<70 years	21 (66%)	77 (64%)	1	42 (71%)	55 (61%)	0.22
≥70 years	11 (34%)	43 (36%)		17 (29%)	36 (39%)	
Sex						
Men	23 (72%)	73 (61%	0.31	35 (59%)	58 (64%)	0.61
Women	9 (28%)	47 (39%)		24 (41%)	33 (36%)	
Baseline ECOG PS score						
0	15 (47%)	67 (57%)	0.32	22 (39%)	59 (66%)	0.0021
1–2	17 (53%)	50 (43%)		35 (61%)	31 (34%)	
CRC localization						
Right	11 (37%)	39 (32%)	0.67	21 (37%)	29 (32%)	0.59
Left	19 (63%)	81 (68%)		36 (63%)	62 (68%)	
Metastases						
Synchronous	23 (74%)	77 (65%)	0.40	48 (83%)	50 (57%)	0.0012
Metachronous	8 (26%)	41 (35%)		10 (17%)	38 (43%)	
Nb of organs with metastases						
1	14 (45%)	47 (39%)	0.55	21 (36%)	39 (43%)	0.49
>1	17 (55%)	73 (61%)		37 (64%)	51 (57%)	
Liver metastases						
Yes	26 (84%)	97 (81%)	0.80	54 (93%)	66 (73%)	0.0025
No	5 (16%)	23 (19%)		4 (7%)	24 (27%)	
RAS status						
Wild type	10 (38%)	30 (31%)	0.49	13 (29%)	26 (34%)	0.69
Mutant	16 (62%)	66 (69%)		32 (71%)	50 (66%)	
B-RAF status						
Wild type	28 (97%)	92 (92%)	0.68	46(92%)	74(95%)	0.71
Mutant	1 (3%)	8 (8%)		4(8%)	4(5%)	
CEA value						
Normal	8 (25%)	36 (31%)	0.66	7 (12%)	37 (42%)	0.0001
>normal	24 (75%)	81 (69%)		51 (88%)	52 (58%)	

Abbreviations: M, men; W, women; CRC, colorectal cancer; PS, performance status.

**Table 2:** Univariate Cox regression analysis for PFS and OS prediction.

Parameters	PFS			OS		
	HR	95% CI	*p*-Value	HR	95% CI	*p*-Value
Age: ≥70 vs. <70 years	1.04	0.74–1.46	0.84	1.08	0.72–1.62	0.71
Sex: W vs. M	0.84	0.6–1.19	0.32	1.28	0.86–1.89	0.22
ECOG PS: 1–2 vs. 0	1.46	1.05–2.05	0.0259	2.66	1.77–3.99	<0.0001
Right vs. left colon	1.07	0.75–1.51	0.72	1.54	1.03–2.31	0.04
Synchronous vs. metachronous mets	0.78	0.55–1.11	0.17	1.24	0.81–1.88	0.32
N of organs with mets: >1 vs. 1	1.21	0.86–1.69	0.27	1.34	0.9–2	0.15
Liver mets vs. no-liver mets	0.9	0.59–1.37	0.62	1.53	0.87–2.69	0.14
CEA: >nal vs. nal	1.04	0.71–1.5	0.85	1.46	0.92–2.32	0.11
RAS: MT vs. WT	0.76	0.51–1.12	0.16	0.71	0.45–1.12	0.14
B-RAF: MT vs. WT	3.27	1.61–6.64	0.001	7.39	3.36–16.25	<0.0001
D_0_-D_28_ CTC kinetics (EPISPOT): Positive at both time points (≥1) vs. other cases	2.52	1.15–5.52	0.0204	2.48	1.14–5.37	0.0219
D_0_-D_28_ CTC kinetics (CellSearch^®^): Positive at both time points (≥3) vs. other cases	3.02	1.45–6.3	0.0031	3.22	1.54–6.74	0.0019

Abbreviations: M, men; W, women; HR, hazard ratio; vs., versus; PS, performance status; nal, normal; mets, metastases; CEA, carcinoembryonic antigen; MT, mutated; WT, wild type; D, day; PFS, progression-free survival; OS, overall survival; CI, confidence interval.

**Table 3:** Multivariate Cox regression analysis for PFS and OS prediction.

Parameters	PFS			OS		
	HR	95% CI	*p*-Value	HR	95% CI	*p*-Value
ECOG PS: 1–2 vs. 0				2.48	1.51–4.07	0.0003
B-RAF: MT vs. WT	3.046	1.43–6.5	0.0043	5.34	2.23–12.79	0.0002
D_0_–D_28_ CTC kinetics (EPISPOT): Positive at both time points (≥1) vs. other cases	2.445	1.04–5.78	0.0414			
D_0_–D_28_ CTC kinetics (CellSearch^®^): Positive at both time points (≥3) vs. other cases	2.461	1.06–5.74	0.037			

Abbreviations: HR, hazard ratio; vs., versus; PS, performance status; MT, mutated; WT, wild type; D, day; PFS, progression-free survival; OS, overall survival; CI, confidence interval.

## Data Availability

The data presented in this study are available in the main article and its supplementary material.

## Supplementary Materials

The following are available online at https://www.mdpi.com/article/10.3390/cancers13122966/s1, Table S1: Patient and tumor characteristics (*n* = 155), Table S2: CTC detection at each time point, Table S3: Prognostic value of CTCs according to the CK19-EPISPOT assay.
