# Supplementary material for: Clinical Relevance of Viable Circulating Tumor Cells in Patients with Metastatic Colorectal Cancer: The COLOSPOT Prospective Study
Source: Cancers (Basel). 2021 Jun 13;13(12):2966. doi: 10.3390/cancers13122966 (PMC8231886; doi:10.3390/cancers13122966)
Supplement: Supplementary file 1 [file cancers-13-02966-s001.zip › cancers-1250037-supplementary.pdf]

cancers MDPI

Supplementary Materials

Clinical Relevance of Viable Circulating Tumor Cells in Patients with Metastatic Colorectal Cancer: The COLOSPOT Prospective Study

Thibault Mazard, Laure Cayrefourcq, Françoise Perriard, Hélène Senellart, Benjamin Linot, Christelle De La Fouchardière, Eric Terrebonne, Eric François, Stéphane Obled, Rosine Guimbaud, Laurent Mineur, Marianne Fonck, Jean-Pierre Daurès, Marc Ychou, Eric Assenat and Catherine Alix-Panabières

Table S1. Patient and tumor characteristics ($n = 155$).

Age	Median = 68y [37–84]
Sex: M / W n(%)	97(63) / 58(37)
Baseline ECOG PS: 0 / 1 / 2 n(%)	83(55) / 59(39) / 10(6)
CRC location: right/left colon n(%)	51(33) / 102(67)
Synchronous/metachronous metastases n(%)	102(68) / 49(32)
Nb of organs with mets: 1 / >1 n(%)	62(41) / 91(59)
Liver mets/no-liver mets n(%)	125(82) / 28(18)
Wild type mutant RAS n(%)	40(32) / 85(68)
Wild type/mutant B-RAF n(%)	123(93) / 9(7)
Normal/ >normal CEA n(%)	44(29) / 108(71)

Abbreviations: M, men; W, women; PS, performance status; Nb, number; mets, metastasis; CEA, carcinoembryonic antigen.

Table S2. CTC detection at each time point.

	CK19-EPISPOT						CellSearch®				
	Total sample nb	≥1 CTCs	≥2 CTCs	≥3 CTCs	Median	[Min-Max]	Total Sample nb	≥1 CTCs	≥3 CTCs	Median	[Min–Max]
D_0	152	32(21%)	18(11.8%)	10(6.6%)	0	[0-14]	150	87(58%)	59(39,3%)	1	[0-302]
D_{14}	149	23(15,4%)	6(4%)	0(0%)	0	[0-2]					
D_{28}	138	17(12,3%)	3(2,2%)	0(0%)	0	[0-2]	138	33(23.9%)	13(9,4%)	0	[0-95]
D_{42}	136	13(9.6%)	2(1.5%)	1(0.7%)	0	[0-7]					
D_{56}	130	15 (11.5%)	5(3.9%)	2(1.5%)	0	[0-28]					

Abbreviations: D, day; CTC, circulating tumor cell.

Table S3. Prognostic value of CTCs according to the CK19-EPISPOT assay.

	PFS			OS		
	**n**	**Median (months)**	***p* (Log Rank)**	**n**	**Median (months)**	***p* (Log Rank)**
			**D_0**			
<1/≥1	120/31	9.8/9	0.21	120/32	26/28.9	0.78
<2/≥2	134/17	9.4/9.4	0.95	134/18	25.1/31	0.84
			**D_{14}**			
<1/≥1	126/22	8.8/9.3	0.47	126/23	25,7/21	0.30
<2/≥2	143/5	8.9/8.5	0.79	143/6	25,3/14,7	0.12
			**D_{28}**			
<1/≥1	121/16	8.2/7.5	0.78	121/17	24/17,5	0.52
<2/≥2	134/3	**8.3/5.8**	**0.008**	135/3	**24.8/10.3**	**0.0003**
			**D_{42}**			
<1/≥1	122/13	7.9/8	0.69	123/13	24.4/20.2	0.54
<2/≥2	133/2	7.9/4.6	0.22	134/2	**24.4/11.3**	**0.04**
			**D_{56}**			
<1/≥1	114/15	7.4/7.6	0.86	115/15	23.9/21.7	0.57
<2/≥2	124/5	7.6/7.3	0.28	125/5	23.9/21.7	0.43

Abbreviations: D, day; PFS, progression-free survival; OS, overall survival.
